# Plant Virus Expression Vector Development: New Perspectives

**DOI:** 10.1155/2014/785382

**Published:** 2014-03-13

**Authors:** Kathleen Hefferon

**Affiliations:** Cornell University, Ithaca, NY 14850, USA

## Abstract

Plant made biologics have elicited much attention over recent years for their potential in assisting those in developing countries who have poor access to modern medicine. Additional applications such as the stockpiling of vaccines against pandemic infectious diseases or potential biological warfare agents are also under investigation. Plant virus expression vectors represent a technology that enables high levels of pharmaceutical proteins to be produced in a very short period of time. Recent advances in research and development have brought about the generation of superior virus expression systems which can be readily delivered to the host plant in a manner that is both efficient and cost effective. This review presents recent innovations in plant virus expression systems and their uses for producing biologics from plants.

## 1. Introduction 

It takes just a quick glance at the number and variety of novel biologics that are currently emerging from the commercial sector to realize that the use of plants as production platforms for vaccines and other therapeutic proteins has over time moved from mere theory and into actual practice. Plant-derived biologics are safe, efficacious, and easy to mass produce. They can offer new solutions to the challenge of providing inexpensive medicines that lack cold chain requirements or an established medical infrastructure for the world's rural poor. While assisting developing countries has always been a fundamental driving force for the development of plant-made biologics, other uses have also been presented and range from facilitating the stockpiling of vaccines against pandemic infectious diseases to the applications of plant-made proteins in the field of personalized medicine [1, 2].

Originally, plant-made biologics were engineered to be expressed from stably transformed plants. As this research area progressed to the point of determining plant-based pharmaceutical performance in actual clinical trials, it became increasingly apparent that lengthy tissue culture and plant regeneration procedures and concerns regarding public perception over the use of GM technology were all major stumbling blocks with regard to the use of transgenic plants that needed to be addressed. The design and implementation of novel transient expression systems through the use of plant virus expression vectors has been one technology that circumvents many of these hurdles. Not only are plant viruses able to produce extremely high levels of foreign protein at low cost and in a matter of a few days postinoculation, but also their use as a technology is more appealing to the public because it lacks the negative connotations that are often associated with GM plants. Indeed, removal of the genes encoding virus movement and/or coat proteins prevents plant viruses from moving from plant to plant and as a result reduces concerns about transmission and cross-contamination of pharmaceutical proteins to weedy relatives.

As research and development of plant virus expression vectors progress, the means of introducing them to the host plant has also advanced. Rather than merely infecting plants with the appropriate virus vector, plant leaves can now be inoculated by agroinfiltration, that is, by incorporating the virus vector into Agrobacterium tumefaciens and infiltrating the leaf using a syringe or a vacuum. Both methods have their own advantages, and both are rapid and scalable and lack the requirement of sophisticated equipment. The universality of these technologies makes them amenable for both commercialization and classroom alike [3].

Over the past decade, plant viruses have been developed both as vectors for biopharmaceutical protein production and also as research tools for plant functional genomics studies, by incorporating virus induced gene silencing (VIGS) as a means to target and downregulate specific host transcripts. The latter technology has great implications for the development of crops with improved characteristics, while the former has the potential to launch a new era for the production of biologics. This review will discuss several of the plant virus expression systems that have been developed to date and will provide selected examples of their application in the field of plant-made biologics.

## 2. Expression Systems Based on Positive Sense RNA Viruses

### 2.1. Tombusviruses

Tobacco mosaic virus (TMV) is one of the most well characterized plant viruses and was the first plant virus examined for vector development. While original TMV-based viral vectors depended on usage of the entire viral genome, second generation vectors were “deconstructed” and consisted of the only sections of genomes that were important for replication; these were subcloned into an assortment of plasmid constructs. The first of these new series of vectors, the MagnICON vector system of ICON Genetics, is a core technology of Dr. Gleba and coworkers [4]. A technique for transfecting plants with these recombinant virus vector modules, known as “magnifection,” involved the infiltration of a suspension of* A. tumefaciens* using a vacuum into all mature leaves of a tobacco plant, thus infecting the whole plant in its entirety [5]. These constructs are part of the T-DNA of a binary vector, and researchers need to merely mix different Agrobacterium strains which harbour these constructs prior to agroinfecting plants. As an example, yields of recombinant human growth hormone protein (hGH) reaching up to 10% of total soluble protein or 1 mg/g of fresh weight leaf biomass have been achieved using this system of expression [5]. Further modifications, including alteration of transcript splicing sites, modification of codon usage patterns, and the introduction of introns into TMV coding sequences, have improved protein expression further.

The technology has been built upon so that it could be incorporated into the Gateway cloning system, a series of plasmids that are used for a wide variety of expression systems [6]. In this case, the need for traditional cloning has been circumvented by a system that involves site-specific recombination. In this way, foreign genes and other DNA fragments alike can be transferred between plasmids to enable the desired recombination event to take place. Protein expression can also be enhanced 10- to 25-fold by the coexpression of the RNA silencing suppressor gene of Tomato bushy stunt virus known as P19. For example, Lindbo coinoculated plants with a TMV-based vector and a viral suppressor of RNA silencing; this culminated in the production of tremendous levels of recombinant protein (between 600 and 1200 micrograms of GFP per gram of infiltrated tissue) after one week postinfection [7].

Other researchers have found that gene expression can be increased several times by placing the foreign gene open reading frame (ORF) closer to the 3′ end of the TMV RNA. This TRBO (TMV RNA-based overexpression) vector lacks the coat protein coding sequence and can produce 100-fold more recombinant protein in plants than the P19-enhanced transient expression system. Lindbo's group found that this vector could generate 100 times more of a variety of recombinant proteins than their P19 silencer system [8].

The number of examples of the use of TMV-based vector technology as a platform for biopharmaceutical production in plants is rapidly increasing. Since this review cannot adequately describe all in the space constructions allowed, only a few are provided in the following section.

The first example illustrates the use of TMV to generate the broadly neutralizing antibody (bnMAb) known as VRCO1 against HIV-1 [9]. VRC01 binds to the CD4-binding site of gp120 and was isolated recently from a slowly progressing HIV-1-infected donor. Broadly neutralizing antibodies are known for their ability to block infection of a wide number of different strains of a particular virus and thus are considered to be used as part of a topical microbicide to block HIV-1 transmission. Monoclonal antibodies are expensive to manufacture on a large scale, and plants offer an attractive alternative for generating biologically active, inexpensive versions of these much needed pharmaceuticals.* Nicotiana benthamiana* plants were used as the host for a TMV vector which contained a full-length version of the immunoglobulin IgG1 from a single polypeptide. This VRCO1 MAb was produced at approximately 150 mg/kg fresh leaf material within days 5–7 postagroinoculation and could be purified on a protein A affinity column. These plant-made antibodies not only were shown to be biologically active in a neutralization assay but also worked synergistically with other microbicides, demonstrating their potential as part of a topically applied microbicide cocktail to act as a prophylactic against infection by HIV-1 [9].

The use of TMV to produce a vaccine for pandemic influenza virus represents the second example. Seasonal influenza virus has become a serious health threat across the globe. New vaccines must be provided every new year to protect against emerging new virus subtypes that circulate every new flu season. The requirement to supply the demand for flu vaccines worldwide is enormous and cannot currently be met. Recently, Petukhova et al. have used a TMV vector to express three different versions of the epitope M2e of influenza virus [10]. The authors used the CP of TMV as a carrier molecule and expressed the epitope as part of a fusion protein that is exposed on the surface of TMV as a nanoparticle. Antibodies raised in mice against the nanoparticle were specific to M2e and mice immunized with the M2e:TMV recombinant virus were resistant to inoculation with lethal doses of influenza H1N1 virus [10].

More extensive investigation into the utilization of TMV as a feasible vector for influenza vaccine production has been pursued by Yusibov et al.'s research group at Fraunhofer USA. The Fraunhofer group has generated a subunit vaccine based on recombinant hemagglutinin from the 2009 pandemic A/California/04/2009 (H1N1) strain of influenza virus [11]. This TMV-based vaccine was demonstrated to be efficacious and free of adverse effects in human clinical trials in the presence or absence of Alhydrogel as an adjuvant, in a manner that is highly comparable with the currently used approved vaccine for H1N1 [12, 13]. This represents the first study that demonstrates a plant produced subunit influenza H1N1 vaccine in healthy adults. In a further study, Neuhaus et al., tested the ability of this tobacco produced vaccine in conjunction with a silica nanoparticle-based drug system to induce an antigen-specific recall response at the site of virus entry in human precision-cut lung slices (PCLS) [14]. The authors demonstrated that the plant produced vaccine was capable of reactivating an established antigen-specific T cell response at the site of virus entry [15].

As a third example, Li et al. have used the TMV-based TRBO vector to express the allergen R8 from dust mites in tobacco plants [16]. The authors used murine asthmatic models to investigate the possibility of using these plant-derived antigens for immunotherapy. The plant-derived antigen behaved the same way as the native antigen, offering the possibility that it might be used in the future for the diagnosis of asthma or the production of a candidate vaccine for allergen-specific immunotherapy of asthma [16].

As a final example, MAGNICON vectors were used to compare expression of the highly unstable recombinant protein, human complement factor 5a (C5a) in tobacco plants [17]. Transient expression of C5a that was subcellular targeted to the ER or vacuole using the MagnICON vector was increased from 0.0003 and 0.001% total soluble protein (TSP) to 0.2 and 0.7% of TSP, respectively, demonstrating the utility of this system to produce biopharmaceutical proteins [17].

### 2.2. Potexviruses

Potato virus X (PVX) and related potexviruses have also been constructed into expression vectors for vaccine production. For example, the L1 protein of canine oral papillomavirus has been expressed in transgenic tobacco chloroplasts using a Potato virus X vector [18]. Similarly, the Human Papillomavirus-16 L2 minor capsid protein has been expressed in plants as part of a fusion protein with the PVX CP [19]. Other potexviruses, including white clover mosaic virus, foxtail mosaic virus, and alternanthera mosaic virus, have also been developed for foreign gene expression. Recently, an expression vector based on plantago asiatica mosaic virus (PlAMV) has been constructed [20]. PlAMV is unique among potexviruses due to an overlap at the third gene of its triple gene block and its CP. As a result, the generation of a recombinant PlAMV expression system resulted in a duplicated CP promoter sequence that is much longer than that generated for other potexviruses. Due to this unusual feature, the PlAMV-based vector was more stable and had the ability to exert a stronger suppression of gene silencing activity than its PVX-based counterpart.

### 2.3. Cucumovirus

The RNA virus Cucumber mosaic virus (CMV) has also been developed extensively for use in the production of plant-made biologics. Although it has a highly diverse host range, CMV's trimeric RNAs are each required for virus infection to be successful and are packaged into icosahedral capsids, thus enforcing a size limitation to the foreign gene that can be inserted. Recently, Hwang et al. constructed the complete tripartite genome on a binary plasmid and replaced the coat protein gene with one for *α*-1-antitrypsin [AAT] [21]. The authors placed one of the protein components of the viral replicase (1a) under the control of the XVE chemically inducible promoter in such a way that recombinant viral amplicons could be induced upon addition of the inducer (*β*-estradiol). This enables all of the components that are required for virus replication to be introduced simultaneously into the same cell, thus improving the efficiency of transgene expression. The authors tried to further improve expression by generating multiple plasmids of reduced size which, when combined, incorporate all of the essential components of the genome that are required for replication. By using both GFP and the* Acidothermus cellulolyticus* endo-1, 4-*β*-glucanase (E1), a cellulose degrading enzyme, the authors demonstrated that this CMV advanced replication system can effectively achieve recombinant proteins at levels comparable to transgenic plants, but without the same time and effort required [21].

CMV has also been utilized as an antigen presentation system to express epitopes of porcine circovirus type 2 (PCV2) capsid protein [22]. Chimeric CMV:PCV2 particles were injected into mice and pigs and analyzed for their ability to induce a PCV specific antibody response. Furthermore, pigs challenged with the virus were able to demonstrate partial protection against infection, suggesting that generation of a plant virus-based vaccine may be a feasible and affordable approach to combat this animal pathogen [22].

## 3. Other Plant Viruses Involved in Optimizing Expression

### 3.1. Cowpea Mosaic Virus

The icosahedral, positive sense RNA virus Cowpea mosaic virus (CPMV) has also been designed for the generation of vaccine and other therapeutic proteins in plants. Initially designed as an epitope presentation system, CPMV chimeric particles have been shown to elicit a robust immune response for a variety of diseases [23]. CPMV has also been used to express entire proteins as fusion products with the capsid protein or movement protein of the virus, which can undergo proteolytic cleavage to release the therapeutic protein.

Medicago, Inc., has also developed their own CPMV vector-based technology which produces virus-like particles (VLPs) carrying influenza virus antigens [24]. These VLPs express a lipid-anchored recombinant HA and are fully protected against lethal viral challenge in both mice and ferrets. This vaccine was placed under further study in a Phase 1 clinical trial of 48 healthy volunteers, who showed that the vaccine was both safe and well tolerated. A Phase 2 clinical trial with over 250 volunteers is now underway. Medicago's production system offers the advantage of inexpensively generating a vaccine within 3 weeks of the release of influenza strain sequence information, with easily adaptable upscaling capacity [24].

Nonreplicating CPMV expression systems have also been developed. These involve the positioning of the foreign gene between the 5′ leader sequence and 3′ untranslated region (UTR) of RNA-2 [25]. Deletion of an in-frame initiation codon found upstream of the main translation initiation site of RNA-2 brought about a substantial increase in foreign protein production. This new series of vectors based on CPMV, known as the pEAQ vectors, have been explored as a means to mass produce large amounts of pharmaceutical proteins in plants without the need for virus replication. Using a high translational efficiency, the Cowpea Mosaic Virus hypertranslational “CPMV-HT” expression system generates extremely high yields of recombinant protein [23]. Many pharmaceutical proteins have been successfully produced in plants using this system, including anti-HIV-1 monoclonal antibodies and influenza A virus vaccines [25]. Recently, Thuenemann et al. used the nonreplicating CPMV system known as CPMV-HT (Hyper-Trans) to generate the ruminant Bluetongue virus- (BTV-) like particles in benthamiana plants. This required the synchronous expression of four distinct capsid proteins in each cell and enabling them to self-assemble into virus-like particles. These plant-derived VLPs were demonstrated to protect sheep against live virus infection [26].

### 3.2. Plant DNA Viruses

Plant DNA viruses, including geminivirus and Cauliflower mosaic virus (CaMV), have also been designed as pharmaceutical protein production platforms. Geminiviruses are small single stranded DNA viruses and are named for their twinned capsid morphology; they infect a broad range of plants and can replicate to extremely high copy numbers. Two geminiviruses have been developed for biopharmaceutical production, Bean yellow dwarf virus (BeYDV), a mastrevirus, and Beet curly top virus, (BCTV), a curtovirus [27, 28]. Both viruses have been constructed in such a way that the replication initiator protein (Rep) is expressed independently from the viral genome. A variety of biologics have been produced using this approach, ranging from a vaccine against hepatitis A virus to a monoclonal antibody against Ebola virus [27, 28].

## 4. Additional Applications for Plant Virus Vectors in Medicine

Besides offering a novel production platform for plant-made biopharmaceuticals, plant viruses have been engineered to provide other medical applications. For example, virus-like particles based on C*owpea mosaic virus *(CPMV) have been designed which can incorporate fluorescent dyes, polyethylene glycol (PEG) polymers, and various targeting moieties on their surfaces for the purpose of creating novel tumour-targeted molecular imaging agents [29, 30]. These CPMV VLPs exhibit high selectivity for molecular targets that are cancer-specific and as a result are effective for in vivo imaging of tumors. CPMV represents an icosahedral nanoparticle and the exterior of its capsid displays 300 accessible lysine residues; each of these can be conjugated to various chemical moieties [30]. Examples of the use of this technology include the construction of CPMV nanoparticles displaying gastrin releasing peptide receptor (GRPR) or vascular endothelial growth factor receptor 1 (VEGFR1) [31].

As another example, Cowpea Chlorotic Mottle Virus (CCMV) has been shown to stably assemble in vitro and package the RNA derived from a mammalian virus, Sindbis virus. The hybrid VLPs were able to deliver and release their RNA contents within the cytoplasm of mammalian cells. The CCMV-based VLP was shown to protect against RNA degradation by cellular nucleases. By conjugating subcellular targeting moieties, these hybrid VLPs could be directed toward distinct sites within the cell [32].

Plant viruses have also been engineered to act as adjuvants to elicit an immune response that is more potent and effective. Recently, nanoparticles that are constructed from the coat protein of the rod-shaped Papaya mosaic virus (PapMV) have been shown to be highly immunogenic and are taken up by dendritic cells [33]. These nanoparticles have been engineered to express an influenza epitope on their surface, and mice and ferrets immunized with these recombinant nanoparticles exhibit an increase in robustness of humoral response to influenza virus infection [34]. TMV has also been demonstrated to stimulate cellular immunity. Incubation of TMV CP fused to T cell tumor-specific epitopes elicited immune responses and protected against tumor challenge in mice, indicating that the virus could act as an antigen carrier and induce an adequate immune response [35].

## 5. Conclusions

The development of plant virus vectors continues to evolve with respect to their ease and breadth of use in the field of plant-made biologics. Improved expression vectors based on deconstructed viruses will most likely remain a trend in the near future. Efforts will be made to address other challenges including enhanced expression levels, the generation of proteins with more complex quaternary structures, and issues concerning host specificity. With the advancement of techniques to facilitate their introduction and transient expression in plants, plant virus expression vectors and components derived from them offer strategic advantages for the rapid and cost-effective production of biologics for the world's poor. The fact that this technology lends itself to other applications as well, including the development of vaccines to combat global pandemics and even cancer targeting therapeutics, offers much needed vigor to address a broadening spectrum of needs for medical researchers for many years to come.
